# Editorial: Impact of dietary factors on human gut microbiota and gastrointestinal endocrinology

**DOI:** 10.3389/fendo.2026.1858858

**Published:** 2026-05-05

**Authors:** Patrik Hansson, Mari C.W. Myhrstad, Lara Costantini

**Affiliations:** 1 Department of Clinical Medicine, UiT The Arctic University of Norway, Tromsø, Norway; 2 Department of Food and Nutrition and Sport Science, University of Gothenburg, Gothenburg, Sweden; 3 Department of Nursing and Health Promotion, Faculty of Health Sciences, Oslo Metropolitan University, Oslo, Norway; 4 Department of Ecological and Biological Sciences, Tuscia University, Viterbo, Italy

**Keywords:** diet, dietary factors, gut microbiome, gut microbiota, inflammation, metabolic health, metabolites, short-chain fatty acids

The recognized role of the gut microbiota in relation to human health and disease development has opened up an expanding and promising research field and has been the focus of a substantial amount of research conducted in recent years. Still, due to its complexity, our knowledge about and understanding of the human microbiome are only in their early phase, encouraging further studies conducted in a broad range of areas. Thus, clinical trials, as well as epidemiological studies, examining how our gut microbiota may be linked to gastrointestinal endocrinology and metabolic diseases, and how it may be affected by external factors such as dietary components and patterns, have high relevance. Dietary fibres are known to be fermented by bacterial strains in the gut, leading to the production of metabolites in the form of short-chain fatty acids (SCFAs) that can impact our metabolic health.

The aim of this Research Topic was to shed light on how dietary factors interact with gut microbiota to influence metabolic health. A total of six original articles, with a large variation of hypotheses and study designs, and one narrative review article have been linked to this Research Topic.

Ohmi-Shimizu et al. investigated the gut microbiota composition in children with phenylketonuria, following a low-protein dietary therapy, compared to their siblings who did not have phenylketonuria.

Suárez-Sánchez et al. examined associations between gut bacterial taxa and nutrient intake as well as changes in body mass index in patients undergoing bariatric surgery.

Chen et al. analysed gut microbiota composition and blood lipids in persons with dyslipidemia compared to healthy controls in a high-altitude Tibetan region.

Fadhillah et al. performed a pilot study on adults with abdominal obesity in Indonesia, examining associations between gut microbiota composition and body composition as well as macronutrient intake.

Chen et al. analysed gut microbiota and metabolites in normal-weight and overweight patients with endometrial cancer and healthy controls.

Wang et al. compared the effects of diets with various macronutrient composition on hepatic metabolism and gut microbiota in mice with metabolic dysfunction-associated steatotic liver disease.

While the findings from these various articles provide us with new information within the gut microbiota field and may serve as inspiration and development of new hypotheses and study designs, more studies with similar aims and study designs are needed to confirm and validate the results.

**Figure 1:**
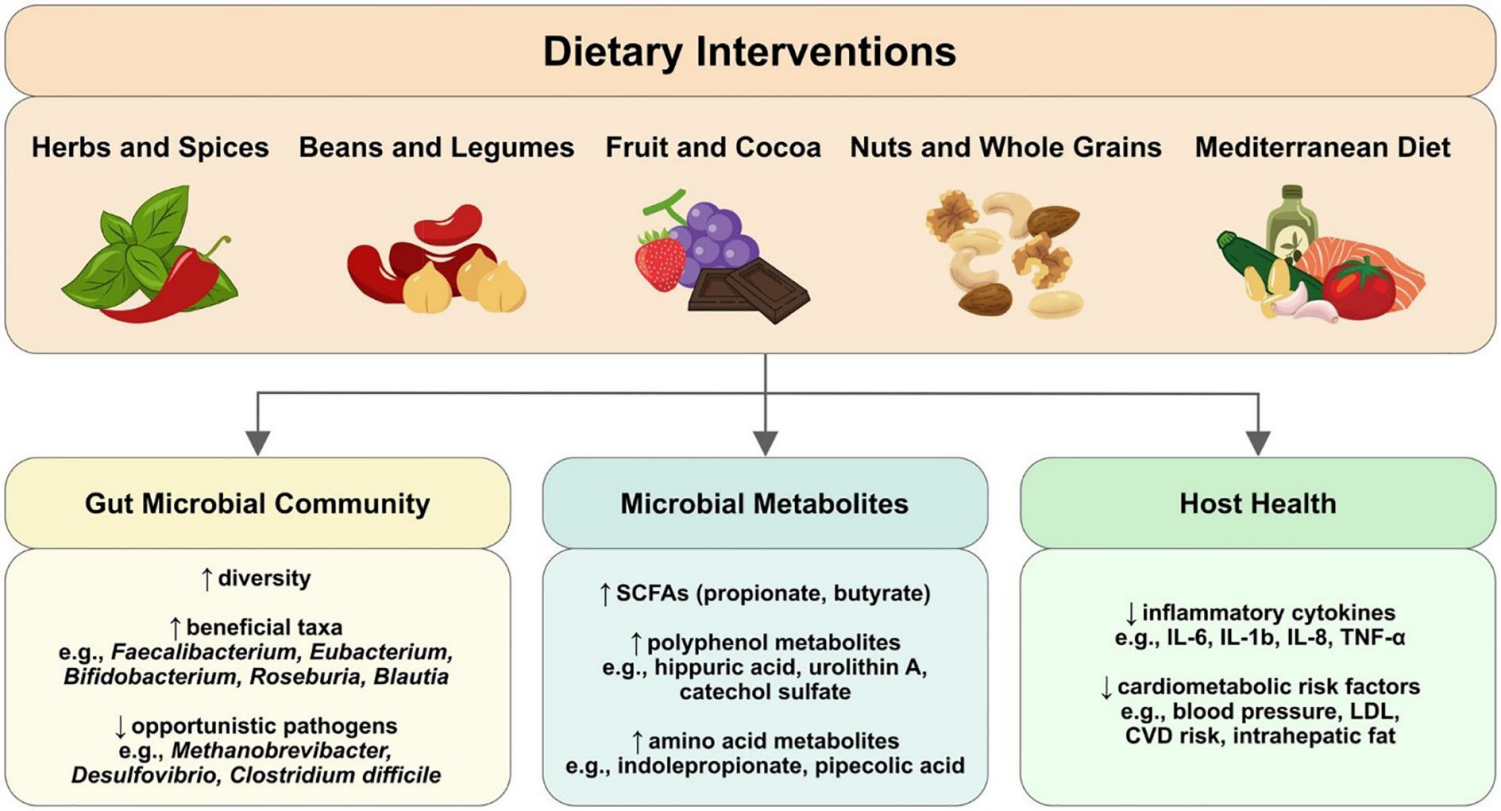
Graphical abstract of Meiners et al. showing the effects of dietary interventions on gut microbial community, microbial metabolites and host health.

The narrative review by Meiners et al. summarized findings from dietary intervention trials conducted in the elderly and individuals with an increased risk for chronic disease. The authors found that even simple dietary modifications, such as the addition of fibres and polyphenol-rich foods, can lead to increased beneficial gut bacteria generating SCFAs. Dietary interventions can also have effects such as increased gut microbial diversity and a reduction in inflammatory markers and cardiometabolic risk factors. An overview of the main findings is shown below in the graphical abstract of their paper (**Figure 1**).

To summarize, this Research Topic highlights the impact of dietary factors on gut microbiota composition, metabolites, inflammatory markers, and cardiometabolic risk factors. It also presents findings from previously unexplored areas, indicating that many hypotheses and objectives yet remain to be investigated and discovered, both in terms of gut microbiota characterization in individuals with different backgrounds and diseases, as well as how various dietary components and patterns are associated with gut microbiota composition, SCFAs, and metabolic health. We hope that this Research Topic will advance current understanding of the interplay between dietary factors, gut microbiota, and gastrointestinal endocrinology, while also encouraging further reflection and future research in this evolving field.

